# Taking ethical photos of children for medical and research purposes in low-resource settings: an exploratory qualitative study

**DOI:** 10.1186/1472-6939-14-27

**Published:** 2013-07-09

**Authors:** Delan Devakumar, Helen Brotherton, Jay Halbert, Andrew Clarke, Audrey Prost, Jennifer Hall

**Affiliations:** 1 UCL Institute for Global Health, 30 Guilford St, WC1N 1EH, London, UK; 2 Royal Hospital for Sick Children, 9 Sciennes Road, Edinburgh EH9 1LF, Scotland; 3 University College London Hospital, 235 Euston Road, London NW1 2BU, UK; 4 Kidasha, Pokhara, Nepal; Lancashire Care NHS Foundation Trust, 31-33 Kenyon Road, Nelson BB9 5SZ, UK

**Keywords:** Photography, Ethics, Informed consent, Teleconference

## Abstract

**Background:**

Photographs are commonly taken of children in medical and research contexts. With the increased availability of photographs through the internet, it is increasingly important to consider their potential for negative consequences and the nature of any consent obtained. In this research we explore the issues around photography in low-resource settings, in particular concentrating on the challenges in gaining informed consent.

**Methods:**

Exploratory qualitative study using focus group discussions involving medical doctors and researchers who are currently working or have recently worked in low-resource settings with children.

**Results:**

Photographs are a valuable resource but photographers need to be mindful of how they are taken and used. Informed consent is needed when taking photographs but there were a number of problems in doing this, such as different concepts of consent, language and literacy barriers and the ability to understand the information. There was no consensus as to the form that the consent should take. Participants thought that while written consent was preferable, the mode of consent should depend on the situation.

**Conclusions:**

Photographs are a valuable but potentially harmful resource, thus informed consent is required but its form may vary by context. We suggest applying a hierarchy of dissemination to gauge how detailed the informed consent should be. Care should be taken not to cause harm, with the rights of the child being the paramount consideration.

## Background

Photographs in clinical practice and research are very common. They can be valuable tools for education, to illustrate a situation, to raise awareness or funding, or to act as evidence and contribute to advocacy. However there are also potential risks to the individual that should be considered. As Franchitto et al. comment when describing the use of medical photography in France, “to take a photograph of a person is to lay bare their identity to the eyes of others” [1].

While healthcare workers have long taken photographs of children, there is now a perfect storm resulting from greater opportunities to work in low-resource settings, an increased number of images from digitalisation, and an increased distribution through the internet and “open access” publishing. This all contributes to a rise in the number of photographs circulating, as well as to the ease and speed with which photographs can be disseminated to large audiences. It is therefore important to consider the implications of these changes for the ethics of photography but there is a paucity of ethical guidelines for the use of images in research [2]. Some medical regulators and international journals require consent to be obtained before taking a clinical photograph, however this standard of regulation is not universal. As specified by the International Committee of Medical Journal Editors [3] “patients have a right to privacy that should not be violated without informed consent” and that this consent should be written. Further “an identifiable patient [should] be shown the manuscript to be published”. Importantly how consent is obtained and the true understanding of the participant is rarely considered. This is particularly important to photographs of children taken in low-resource settings who can be more vulnerable to misrepresentation. Disparities in the power relationship between the photographer and the subject are often increased in medical and research settings and these may be further exacerbated in low-resource settings and where there are language and cultural differences.

We used qualitative methods to explore the importance of taking an “ethical approach” to photographing children in an overseas medical or research setting. In particular, we concentrated on what informed consent means in this setting and discuss the challenges in achieving this. In the discussions we do not include photographs that are part of the direct clinical management of a patient that are kept only in a patient’s notes, for example those of the response of a rash to treatment over time. The discussions cover all other photographs in medical and research situations.

## Method

We conducted three focus group discussions to explore the ethical issues surrounding photographing children in medical or research contexts in low-resource settings. We used focus groups to enable more nuanced discussions so that participants could talk about their experiences and opinions amongst each other rather than in response to interviewer questioning. The participants described their own experiences of taking photographs and of witnessing photographs being taken. On occasion, such as when talking about advocacy purposes within the media, participants voiced their own opinions on the subject. The participants had not taken photographs to be used for advocacy purposes.

We sampled participants from three sets of professionals with similar levels of experience: Group1, researchers from University College London (UCL) with overseas experience in child health; Group 2 paediatricians who had worked in the Voluntary Service Overseas scheme run in conjunction with the Royal College of Paediatrics and Child Health; and Group 3 doctors who had studied International Health at UCL. Group 1 was made up of non-medical researchers. All bar one member of this group had the majority of their overseas research experience in South Asia (India and Nepal). The other member of this group’s main experience was from Malawi. For three of the five people, this research formed their PhD including multiple trips over three to four years. For the other two members, this was from research projects over a continuous two month period. Groups 2 and 3 were made up of medical doctors only. Group 2 participants were all senior paediatric trainees who had spent one year based in the country, one in Sierra Leone, one in The Gambia and one in Cambodia. Group 3 was more mixed with placements from a few weeks to months doing medical work, research and internships/ projects with non-governmental organisations. Their main experience was from India, Romania, Tanzania, Thailand and Zambia. One participant had also spent time working in Peru. We aimed to have between five and eight people in a group. Unfortunately this number was not achieved in the second group, however based on the content and quality of the discussion we felt that findings from the second group were still relevant [4].

The focus groups were held by teleconference using Skype software version 5 (Microsoft, Washington, USA). Video conferencing was not possible due to the low bandwidth of some participants. A trained facilitator (DD) coordinated the focus groups following a topic guide that covered issues such as the experience of taking photographs of children and the meaning of informed consent. The focus groups were recorded with Ecamm Call Recorder version 2.3.24 and transcribed. NVivo software, version 10 (QSR International, Melbourne, Australia) was used for analysis. We used the Framework approach to analyse the qualitative data [5]. The transcripts were reviewed independently by two authors (DD and JAH) using codes that were developed from the original study objectives and refined during the indexing of the data. We discussed the application of codes until consensus was agreed. The codes were interpreted according to the research objectives and new themes emerging from the data. We assumed that the discussions in each group would cover different issues, so the groups were initially analysed separately. However we did not find that they were very different so the results were combined allowing more in-depth analysis.

The UCL research ethics board was contacted during the conception of the project. The board’s policy was that, as the study participants were key informants based in the UK, the study posed minimal risk and could proceed without ethical approval. All participants consented to take part and for their comments to be published anonymously.

## Results

There were 13 participants in the focus groups: eleven women and two men. There was no significant difference in hierarchy within the groups and all members were relatively junior in their careers. All had worked overseas recently and had experience of digital cameras, filming, and common internet social media and photo sharing sites. All the participants were currently based in the UK but several, particularly those doing research, divided their time with work overseas. The participants had a wide range of experience in low-resource settings, predominantly in Africa and South Asia. We acknowledge that experience from the Americas was lacking. Two people were themselves parents and gave insight into a parental perspective on this issue.

### Value of photos

In all three groups most participants thought there is value in taking photographs, even describing them as ‘the most powerful tool I have to hand’ [Group-1 participant-1]. The discussion began with the participants describing why photographs are taken, as summarised in “Main reasons for taking photographs” below. A clear distinction was made between how someone would approach taking a photograph when used for teaching or research purposes and when they are taking ‘personal’ photographs as tourist in a country. The latter being more akin to a journalist. Photographs of the environment were generally used to set the scene when describing activities overseas, often in presentations. The further discussions generally did not refer to either personal or environment photographs.

Main reasons for taking photographs

1. Teaching Photographs were of use in demonstrating clinical signs to medical students or other clinicians. It was acknowledged that there were a multitude of potential uses for these types of photographs, from small group teaching through large group lectures to publication in a journal or textbook which might include being published on the internet.

2. Research There were several instances of photographs being used as data in research. They could also be used to feedback the findings of research to the communities that had participated in it.

3. Environment Sometimes photographs could be useful simply to show the environment to those who have not been there or provide context. They could be of living conditions in a different country or of the standard of infrastructure and cleanliness of a health facility.

4. Personal Participants were clear that there was a distinction between the kind of photos that you might take as a tourist and those that you would take as a clinician.

5. Advocacy The value of photographs was especially apparent when being used for advocacy purposes.

Photographs of children were considered an important subset of all photographs taken: “I think that the sensitivity about children is that they often make such great photos in terms of a child not looking very well in research” [Group-1 participant-2]. Photographs were seen as being able to ‘say far more than a group of demographic statistics’ [Group-1 participant-1]. Participants also noted the usefulness of photographs for advocacy: ‘it’s important to have a human face to things … [or] you wouldn’t have an emotional response’ [Group-3 participant-1]. Some participants wondered ‘how necessary it often is’ to take photographs [Group-1 participant-2] and whether taking pictures was ‘simply out of voyeurism’ [Group-3 participant-1]. Many described unease in taking the photographs, and their concerns were most commonly related to the issue of consent. One participant commented that ‘I was looking earlier on at a … picture of a child crying and I can see exactly why they are using it and why they would need to use it. But I can’t get away from the fact that if that was me, I wouldn’t want a photo of myself used in that way’ [Group-3 participant-2]. One participant, who was also a parent, said ‘I really respond really strongly to the thought of someone putting a picture of … my daughter, on Facebook. I just really don’t feel that people should be having access to pictures of her’ [Group-3 participant-3]. It is noteworthy that none of the participants had been refused consent to take a photograph. On the contrary, many reported situations where people, and particularly children, were keen to have their photo taken.

### Consent

**Figure 1:**
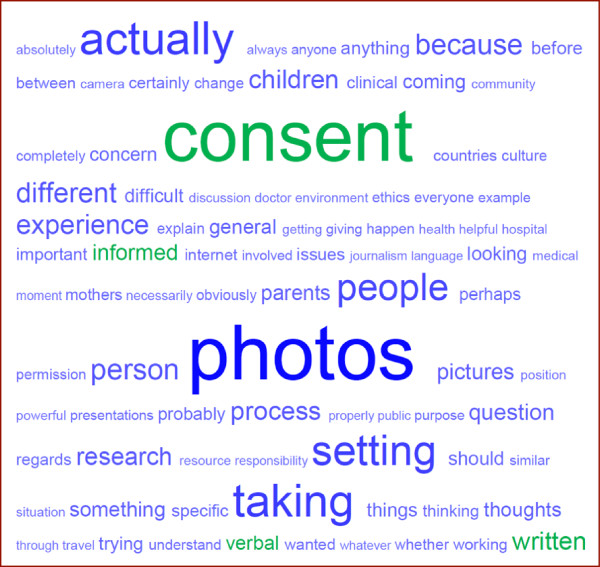
**Word cloud showing the most common words used in the focus groups.** Created using NVivo software, version 10 (QSR International, Melbourne, Australia).

The overwhelming message from all focus groups was the need for consent when taking photographs, particularly of children. However participants also highlighted difficulties in doing this. The word cloud in Figure 1 shows the most frequent words used during discussions, with the size of the word representing its frequency. Consent and its equivalents was the most frequently used word (n=206) after photographs and its equivalents (n=244).

Several barriers to informed consent were described:

### Differing concepts of consent

Participants described situations where there was no concept of informed consent, and where patients ‘looked at me as though I was absolutely daft trying to get their consent’ [Group-2 participant-2]. Some thought that consent was a “Western” value, ‘In the West we have got very much a culture of individualism with individual rights to consent or dissent to everything. Whereas other cultures don’t necessarily have that culture of individualism’ [Group-2 participant-2]. Identifying who should give consent also posed challenges: ‘Asking the translator to ask the child, and I am talking to an 8, 9, 10 year old, and not asking the parent, culturally that just didn’t compute’ [Group-2 participant-2]. Most participants asked the parent or guardian in addition to the child, and the issue of children’s competence to give consent was raised briefly. Generally, even when asking the child, the parent would respond.

### Language

Many participants reported language barriers. Sometimes no interpreter was available and participants reverted to a ‘kind of a body language consent type thing’ which involved making ‘a few signs and show[ing] a camera’ [Group-3 participant-1], or ‘colleagues … would say yes, yes, yes, it’s fine to take pictures’ [Group-1 participant-3]. Others used interpreters but expressed doubts as to the validity of this, especially where ‘it would involve several translators down each tribal language’ [Group-2 participant-1] meaning that ‘quite what was at the end of the chain I’ll never know’ Group-2 participant-2]. There were also times when the words didn’t exist in the other language [Group-2 participant-2]. Only one participant reported having no language problems [Group-3 participant-4].

### Understanding

Many participants were concerned that community members or patients had insufficient knowledge of the potential uses of the photographs and who might see them. One participant mentioned having worked in a location where ‘most [people] have never seen a camera’ [Group-3 participant 4] or where ‘most people have never used a computer at all and had never heard of e-mail and didn’t know anything about publications and had never seen any journals or textbooks’ [Group-2 participant-3]. Patients therefore had ‘no conception of how wide the distribution of these photos could be’ [Group-3 participant-3] leading one participant to comment ‘any attempt to get written or even verbal proper informed consent would just be a joke. It would just be a pretence, thinking that they understood the implications. It would be far too complex’ [Group-2 participant-3].

There was a sense that the internet had already changed the way media such as photographs were used, and that in the future ‘photos and other media like video are going to become more relevant’ [Group-1 participant-4], posing a challenge as to ‘how we can actually achieve this concept of informed consent in today’s kind of world of media’ [Group-3 participant-2].

### Type of consent

Participants thought that the type of consent required varied according to the use of the photograph: “I think really focusing on what you are going to use this photo for will inform the consent process that you will use in the field” [Group-1 participant-1]. They often mentioned the practicalities of obtaining consent when taking a photograph and gaining consent for group photographs was considered particularly difficult. One participant mentioned getting consent from the nurses on a ward. “I guess we took a general photo of the ward. But then you would have to walk round and check with everyone. I guess it is easier just to check with the nurses.” [Group-2 participant-1].

Notwithstanding the difficulties in gaining informed consent it was recognised that consent could be verbal or written or non-verbal (as described above). Whilst written consent was felt to be ‘preferable’ [Group-1 participant-5 and Group-3 participant-4] or ‘ideal’ [Group-1 participant-4] there was some uncertainty that it was practically achievable in the field [Group-1 participant-4]. Thumbprints or an X were also acknowledged but were seen to be ‘fairly meaningless if you think about ever trying to chase it up or prove it’ [Group-2 participant-2]. There was also the concern that written consent would be a veneer for inadequately obtained informed consent.

Most participants had only gained verbal consent, with the exception of the research context where written consent was obtained. Reasons cited for this included the oral culture and problems with translation and literacy. Some countries had ‘very much an oral, … voice culture’ [Group-2 participant-1] and therefore ‘probably verbal consent would be enough and that was us bringing English values into it’ [Group-2 participant-1]. In some participants’ experience there was an active distrust of written consent due to bad experiences with, or suspicion of bureaucracy, which made verbal consent preferable. Overall it was thought that the choice between written or verbal consent ‘should depend on the circumstances’ [Group-1 participant-2].

In discussions about who benefited from the consent process, several participants reflected that written consent was the standard in the UK for medico-legal purposes and protection of the photographer rather than being beneficial to the patient.

### Power disparity

All groups raised the issue of power disparities, commenting on the ‘unequal power relation’ between the photographer and those being photographed [Group-1 participant-3]. This could occur either in the doctor-patient relationship or in the NGO–community relationship. Participants were concerned that people did not feel free to refuse in these situations and could be taken advantage of.

### Teleconference focus group discussions

The use of teleconferences for focus group discussions is rare in qualitative research. While there are challenges, this method does enable inclusion of remotely situated participants and facilitates discussion that would otherwise be impossible. Difficulties included variable internet connection, maintaining a flow of conversation without non-verbal cues and domestic distractions interrupting the conversation and requiring participants to temporarily leave the conversation. The lack of visual cues was a significant limitation although it appeared that when participants knew each other these were less important.

## Discussion

All groups reiterated that photographs of children in medical and research settings are useful because they enrich teaching, research, and advocacy, and in many cases are essential to them. Most thought that children themselves were generally happy to have their photograph taken although some participants reflected that despite this the children may be portrayed in a manner which the participants were uncomfortable with.

Despite the importance of photographs, participants agreed unanimously that they can potentially have negative consequences for the subjects. Informed consent was deemed especially important when children featured in photographs and where power imbalances influence the ability to refuse the photographer. This disparity in power is not unique to low-resource settings but is more pronounced. Our experience and that of colleagues, working in NGOs suggests that the safety and privacy of children, particularly those at risk, has been compromised by photographs that identify them and their location, due to the images not being adequately protected and re-surfacing in the public press. This was a concern of the focus group participants, especially the ones with children who identified that they would not be comfortable having images of their own children available on the internet. On other occasions children and their families have been cited in photographs as having a stigmatizing condition, such as HIV. In instances where medical photographs are used for fundraising or advocacy purposes there may be conflict between protecting the privacy and dignity of the child and achieving the aims of the organisation. Being mindful of the rights and dignity of the child in the photograph should help protect against exploitation but there is a danger that once the image is available on the internet it can be taken out of context. This suggests the importance of regarding photographic images with the same degree of care and rigor that might be applied to other forms of data and taking appropriate steps to protect their storage and use.

### Consent

Overall there was consensus that consent is required but views varied about how to obtain it, by whom, from whom, and when. Obtaining full informed consent was considered the biggest challenge to “ethical photography”. The meaning of informed consent seemed to vary from a relaxed interpretation to full disclosure of risks and implications. Some even thought fully informed consent is impossible. The view expressed that it was easier to check with the nurses may not be uncommon. But unless the health workers have parental or guardian responsibility for the child, then this approach fails to address the issues of consent and suggests more about photographer convenience. According to Mackintosh, informed consent “requires that subjects be competent to make a decision about their image, are adequately informed about its use, comprehend what is being communicated, and give voluntary consent to having their picture taken and its subsequent use” [6]. Achieving all these aspects of consent is difficult, particularly in contexts where poor or marginalized groups are not commonly asked for consent on any issue. Whilst not explored in depth by the groups, the issue and validity of children’s assent (either in the presence of parental consent or not) is also particularly pertinent to low resource countries where many minors are in settings without parental care or are caring for others. It can be argued that where a child is competent, then their consent alone should be sought. In any case, as commented by Pink “consent does not give researchers the right to use images in an unrestricted way” [7].

A common hindrance to obtaining consent was the language barrier. Interpreters were often used but this is open to misunderstanding or miscommunication. Written consent was generally thought to be preferable but problematic. We do not feel that this is necessary in all occasions. According to Banks, oral consent is sufficient when the participant has a low level of literacy [8]. In this exploratory study, most participants thought written consent was for the benefit of health practitioners or researchers, to defend themselves for medico-legal purposes.

A conclusion from the groups was that any consent procedure should be appropriate to the setting. This is supported by research that reiterates the need for ethical challenges to be considered within the context in which they occur [9]. Between and within a country, understanding may vary greatly and there may be different people from whom consent should be taken. For example in some societies a sibling, grandparent or co-wife may accompany a child to seek medical care and represent the family. It was also stressed that issues such as informed consent and individual rights are not considered important in every setting. It may be deemed that collective decision-making or approval from an elder relative is more important. In a relativist sense, the type of consent gained may need to vary according to cultural or physical practicalities as well as being determined by the proposed use of the photograph. It was also stressed that care should be taken to avoid imposing what was described as “Western values” (e.g. an emphasis on individual rights and consent) on others, although it could be argued that awareness and demand for the practical realisation of human rights is growing in many settings. As stated in the groups, many people are uncomfortable when asked for consent because it is not customary for this to happen. However seeking consent is seen as key in many countries as part of increasing the demand for social equity and empowerment. If our actions ignore that, then we reinforce the hierarchical status quo. As healthcare workers we are advocates for the child and have a responsibility to protect the dignity and rights of the child, both within our own setting and in the global arena.

All groups mentioned the importance of recent advances in media technology. Even in low-resource settings it is common to see digital cameras and camera phones. The internet, in particular social networking sites, make photographs available around the world. This enables the image to be taken out of context, the child misrepresented or the image digitally manipulated [10]. The photographer loses control over the photo and many websites acquire rights to the photo when it is uploaded. The importance of context was also stressed here in that in some settings a computer is an alien concept while in others people regularly update their social network status.

In general, ownership of photographs resides with the person taking the photograph, not the subject. However for medical images the person taking the photograph does not necessarily own the copyright. In the UK the copyright of medical images is owned by the health authority on behalf of the Secretary of State but in other countries the person taking the photograph owns the copyright. The copyright for images published in scientific journals tends to reside with the publishing group although with the rise in open-access publishing there has been a shift towards authors retaining more rights. Large publishing groups, such as BMJ Publishing groups Ltd, recommend that “Images can be downloaded and used for private study, but must not be copied, published, resold or used for public lectures without permission” although there is no formal regulation or governance to prevent the unauthorised use of photographs [11].

There is a dearth of information about ownership and copyright of photographs in low resource countries which, combined with an evolving globalized digital media and lack of copyright regulation, makes it even more pressing to consider the ethical implications of photographing children in low resource settings.

### Current guidelines

The United Nations “Convention on the Rights of the Child” (UNCRC) [12] and “Reporting guidelines to protect at-risk children” [13] provide internationally agreed frameworks that should underpin policy and decision-making in this area. Within the UNCRC, specific articles about the rights of children to participate in decisions that affect them, to be protected, and for all actions to be taken in their best interests are prominent; these principles apply equally to taking photographs of children. Photographs are used with best intentions to advocate for ‘greater good’ but those that misrepresent or cause additional vulnerability to the child contravene the Convention.

The UK General Medical Council (GMC) has recently set out guidelines for recordings of patients that both form part of their care and for secondary purposes, such as teaching, as shown in “Principles of the GMC guidance on recordings” below [14]. They stress the need for informed consent for visual and audio recordings, including photographs. Written consent is recommended, especially when publishing in the media, but is not mandatory. Many medical journals also have guidelines requiring written consent. The photos should be anonymised and where children have the capacity they should be asked for their assent (as a minimum) to be photographed in addition to their parent/guardian [14]. The GMC however only has jurisdiction over British medical doctors and not other health workers. Also, while this guidance is explicit about the need for consent, it is slightly ambiguous as to what “informed” consent actually constitutes: “give patients the information they want, or need, about the purpose of the recording”. Furthermore, universal guidelines are unlikely to be applicable in all settings; guidelines should therefore be able to be adapted according to the setting and should be periodically reviewed to ensure their on-going relevance.

### Principles of the GMC guidance on recordings [14]

“When making or using recordings you must respect patients’ privacy and dignity, and their right to make or participate in decisions that affect them. This means that you must:

•give patients the information they want, or need, about the purpose of the recording

•make recordings only where you have appropriate consent or other valid authority for doing so

•ensure that patients are under no pressure to give their consent for the recording to be made

•where practicable, stop the recording if the patient asks you to, or if it is having an adverse effect on the consultation or treatment

•anonymise or code recordings before using or disclosing them for a secondary purpose, if this is practicable and will serve the purpose

•disclose or use recordings from which patients may be identifiable only with consent or other valid authority for doing so

•make appropriate secure arrangements for storing recordings

•be familiar with, and follow, the law and local guidance and procedures that apply where you work.”

### Recommendations

We advocate using guidelines such as those mentioned when taking a photograph and for its subsequent protective storage, but acknowledge that adhering to the guidelines in low-resource settings is complicated. Training for health professionals in the ‘real-life’ application of such guidelines, particularly in the context of a more practical understanding of the UNCRC, would improve adherence.

As noted, not all journals have guidelines on the requirement for informed consent for the use of photographs. This should be standardised and, given the additional complexities involved, particular attention should be given to the use of photographs of children and/or from lower income countries.

Following on from the findings of this study and research documented within the Economic and Social Research Council review on ethical issues in visual research [15], we suggest that informed consent is gained in all cases in which photographs are taken and propose a “Ladder of Dissemination” that helps researchers to articulate just how widely photographs may be disseminated in order for them to be explicit about this while seeking informed consent and describing the process of consent-seeking in presentations and publications (Table 1). Photographs that are freely available on the internet would have the highest degree of dissemination and require careful and exhaustive informed consent detailing potential uses and distributions. Whilst not obviating the necessity for informed consent, a photograph that goes no further than someone’s personal archive or is used for small group teaching would have less stringent criteria. As discussed in greater detail by Mavroforou et al. [16] the question of images of non-identifiable parts of a child’s body is complicated. We feel that such photographs would require informed consent, especially where greater dissemination is possible. Where the degree of truly informed consent achieved from a child or parent is questionable, we suggest moving to a lower level of dissemination, anonymising or not taking the photograph.

**Table 1:** Ladder of dissemination

**Level**	**The degree to which the photo will be disseminated**
**1**	The photo will be used for small audience teaching or will reside in a personal archive.
**2**	The photo will be used in large audience presentations or teaching.
**3**	The photo will be published in journals, textbooks or the internet and will be available to a limited audience with copyright restrictions.
**4**	The photo will be published in journals, textbooks or the internet and will be available to anyone, but copyright restrictions apply limiting its onward use.
**5**	The photo will be freely available on the internet.

### Limitations

The main limitation of this study was that all the participants were clinicians and researchers residing in the UK with research affiliations linked to one university. We do not have the opinions of healthcare workers or researchers who reside in low-resource settings and, most importantly, of parents and children in these settings. In this respect we see this work as only the beginning of a process and we call for more research in this subject in different parts of the world.

## Conclusions

All participants in our exploratory study acknowledged the importance of photographs of children in medical and research settings in low-resource settings, but also highlighted potential challenges. The risk from photographs is generally minimal but not insignificant, especially in a digital era, and for some children can result in serious consequences. Informed consent was deemed paramount but the method through which this is obtained can vary in different contexts. We would encourage further training on this subject and endorse the application of guidelines such as those set out by the GMC. Extrapolating from this, we suggest using the ‘Ladder of Dissemination’ as a tool to gauge how detailed the informed consent should be and help maintain the rights of the child being photographed.

## Competing interests

AC, DD, HB and JH are members of the International Child Health Group (ICHG) executive committee that is helping to draft guidelines on ethical photography of children within the ICHG and Royal College of Paediatrics and Child Health. This study is separate to the guideline development.

## Authors’ contributions

DD, HB and JH designed the focus group topic guides. DD ran the focus groups, with HB recording and taking notes. DD, HB, JH and JAH transcribed the focus group recordings. DD and JAH analysed the qualitative data. The paper was drafted by DD, HB and JAH with comments and amendments by all other authors. All authors read and approved the final manuscript.

## Pre-publication history

The pre-publication history for this paper can be accessed here:

http://www.biomedcentral.com/1472-6939/14/27/prepub
